# Keeping a Completely Autotrophic Nitrogen Removal over Nitrite System Effective in Treating Low Ammonium Wastewater by Adopting an Alternative Low and High Ammonium Influent Regime

**DOI:** 10.1155/2018/9536761

**Published:** 2018-04-17

**Authors:** Qinglong Chang, Weigang Wang, Jie Chen, Yayi Wang

**Affiliations:** State Key Laboratory of Pollution Control and Resources Reuse, College of Environmental Science and Engineering, Tongji University, Siping Road, Shanghai 200092, China

## Abstract

An alternative low and high ammonium influent regime was proposed and adopted to keep a completely autotrophic nitrogen removal over nitrite (CANON) effective when treating low ammonium wastewater. Results show that, by cyclic operating at an alternative low and high ammonium concentration for 10 days and 28 days, the CANON system could effectively treat low ammonium wastewater. Excessive proliferation of nitrite oxidizing bacteria (NOB) under low ammonium environment was still the challenge for the stable CANON operation; but with 28 days of a high ammonium treatment combined with a sludge retention time control, the NOB overproliferated in the low ammonium operational period could be under control. Specifically, when the nitrite oxidation rate reached 8 g N/m^3^/h, the CANON system should enter the high ammonium influent operating mode. 16S rDNA high-throughput sequencing results show that the appropriate sludge discharging provided an environment favoring* Candidatus* Jettenia.

## 1. Introduction

Anaerobic ammonium oxidation (anammox) is a new pathway of autotrophic biological nitrogen removal for wastewater treatment with the advantages of less aeration consumption, low biomass production, and carbon savings [1, 2]. Complete autotrophic nitrogen removal over nitrite (CANON) system, which combines anammox with partial nitrification in a single reactor, is one of the most promising methods to achieve energy neutral or even positive in wastewater treatment plants (WWTPs) [3, 4]. In this process, ammonium is first partially oxidized to nitrite by aerobic ammonium oxidizing bacteria (AOB) through controlling dissolved oxygen (DO) at a low concentration. The residual ammonium is oxidized to nitrogen gas with the generated nitrite by anammox bacteria subsequently [5, 6]. Compared with the traditional nitrification and denitrification systems, CANON process can be a more promising and economic technology for wastewater treatment because of 63% less oxygen consumption, no requirement of biodegradable organic carbon addition, and less N_2_O emissions [5, 6].

So far, CANON systems have been mostly applied to treat high ammonium wastewaters, such as landfill leachate, swine wastewater, and reject water in WWTPs (known as sidestream anammox) [7–12]. This is because high ammonia (as well as high free ammonia (FA)), high temperature, and a low DO control favor selecting both AOB and anammox bacteria as the dominant functional microorganisms, thus maintaining the stable partial nitrification and anammox processes. Specifically, the physiological differences between AOB and nitrite oxidizing bacteria (NOB) should be considered; that is, environmental factors such as pH, temperature, FA, free nitrous acid (FNA), and DO must be controlled favoring AOB proliferation in the partial nitritation step to produce an anammox-suited substrate [13].

When treating mainstream wastewater that is characterized with low ammonium and ambient temperature, stressing NOB and maintaining the activity of anammox bacteria are the two key factors determining the CANON performance [14]. Indeed, substantial efforts have been made to optimize the treatment process for a steady performance in CANON systems when treating mainstream wastewater. Han et al. [15] used screens to separate flocs and granules in mainstream deammonification to wash out NOB, and they found that over 80% of NOB was washed out and up to 70% of nitrogen removal efficiencies was achieved. Similarly, Malovanyy et al. [14] tried integrated fixed film activated sludge reactor for nitrogen removals from municipal wastewater using a deammonification process; by intermittent aeration with a high DO set-point (1.5 mg/L) and decreasing nonaerated time (30 min), NOB was successfully out-selected. However, other effective and operative approaches to ensure the effective nitrogen removal performance of CANON systems are still urgently required especially when treating low ammonium wastewater.

In this study, a novel operational pattern for CANON system treating low ammonium wastewater was developed by alternatively inflowing low ammonium concentration (mimic mainstream wastewater) and high ammonium concentration (mimic sidestream wastewater). The influent exchange frequency and the key controlling factors for effective CANON operation were examined. To elucidate the microbial structure of functional bacteria and their influence to the nitrogen removal performance, the composition of the bacterial communities in the CANON system during operational period was analyzed by 16S rDNA high-throughput sequencing technology. The proposed operational strategy hopes to help CANON processes to be effectively applied in municipal wastewater treatment.

## 2. Materials and Methods

### 2.1. Reactor Configuration

A lab-scale sequencing batch reactor (SBR) with a working volume of 2.5 L was used in this study. The SBR was set in a thermostatic water bath to keep temperature at 30°C. The experimental biomass in the reactor was mixed by a propeller stirrer at 100 rpm. An aeration device linked with an air pump was fixed at the bottom of the SBR to provide oxygen for partial nitrification. Air was regularly supplied to the SBR by an air pump.

### 2.2. Operational Strategies

**Figure 1:**
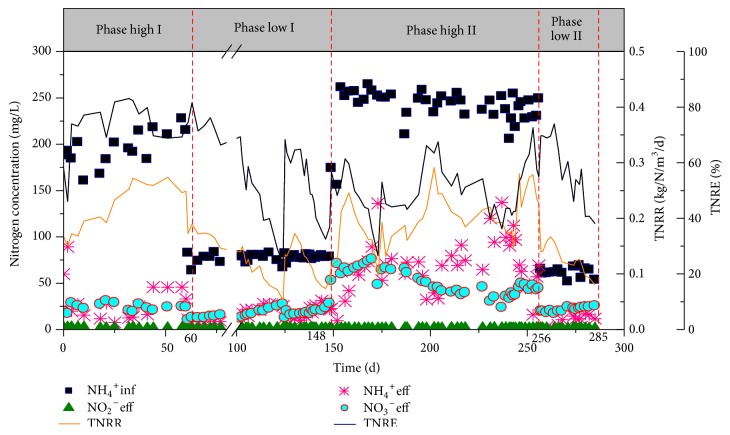
Profiles of nitrogen compounds (expressed as influent ammonium concentration, effluent ammonium concentration, effluent nitrite concentration, and effluent nitrate concentration), total nitrogen removal rate (TNRR), and total nitrogen removal efficiency (TNRE) of the CANON system over 285 days of operation.

An alternative low and high ammonium influent regime was adopted in the experiment. As shown in Figure 1, the whole experimental period was divided into four operational phases according to the influent ammonium concentration: Phase high I (0–60 d), Phase low I (61–148 d), Phase high II (149–256 d), and Phase low II (257–285 d). Phases high I and II were operated at a high ammonium concentration (approximately 240 mg NH_4_^+^-N/L) while Phases low I and II (approximately 61 mg NH_4_^+^-N/L) were operated at low ammonium concentration. A typical SBR cycle lasts for 4 h or 8 h, respectively, at low or high influent ammonium concentrations (Table 1). By operating in an alternative low and high ammonium influent regime, the NOB inhibition and the dominance of AOB and anammox bacteria in the CANON system can be ensured.

### 2.3. Seed Sludge and Feeding Medium

The seeding granular sludge (2.5 L) for the experimental CANON system was from another CANON system treating high ammonium saline wastewater in our laboratory (Figure S1(A)) [3].

Synthetic wastewater was continuously introduced into the reactor using a peristaltic pump in the inflow period and the pump was controlled by a liquid level controller to control the water volume of 2.5 L. The composition of the synthetic wastewater was KH_2_PO_4_ 0.05 g/L, CaCl_2_ 0.3 g/L, MgSO_4_·7H_2_O 0.3 g/L, NaHCO_3_ 1.25 g/L, FeSO_4_·7H_2_O 0.00625 g/L, Na_2_EDTA 0.00625 g/L, and 1.25 mL/L of trace elements solution. The composition of the trace elements solution was prepared according to Lotti et al. [16]. NH_4_Cl was added to the feeding medium to reach a desired ammonium concentration in the influent. The pH was maintained at 8.0 at the beginning of each cycle by adding hydrochloric acid solution (0.2 M) or sodium hydroxide solution (0.2 M).

### 2.4. Analytical Methods

Water samples were collected and stored in a 4°C refrigerator until analysis. The concentrations of NH_4_^+^-N, NO_2_^−^-N, and NO_3_^−^-N were measured regularly according to the standard methods [17]. The DO concentration and pH were detected using online probes (WTW Multi350i, Germany).

### 2.5. Sample Collection, DNA Extraction, and PCR Amplification

Sludge samples were collected from the CANON system on days 60, 79, 124, 125, 148, 257, and 285 (the days before the change in the operating condition) and stored at −20°C after centrifugation. Total genomic DNA was extracted in triplicate from each sample using the Power Soil DNA Isolation Kit (Sangon, China) according to the manufacturer's instructions. The quality of the obtained genomic DNA was examined by 1% (w/v) agarose gel electrophoresis and concentration measured with NanoDrop spectrophotometer 2000 (Thermo Scientific, USA).

Polymerase chain reaction amplification of the V3-V4 region of the 16S rDNA gene was then conducted using primers 341F (5′-CCTACACGACGCTCTTCCGATCTN-3′) and 805R (5′-GACTACHVGGGTATCTAATCC-3′) with the reverse primer containing 6-bp barcodes to tag each sample (Majorbio Bio-Pharm Technology Co., Ltd., Shanghai China). PCR amplification and 16S rDNA high-throughput sequencing were performed according to our previous study [3].

## 3. Results and Discussion

### 3.1. The Stabilization of the CANON System at High Influent Ammonium Concentration

The CANON system operated for 285 days, and the long-term nitrogen removal performance was shown in Figure 1. The biomass was initially cultivated with high ammonium concentration (196 ± 18 mg/L) to set up the CANON system (Figure 1, Phase high I). After acclimation for 32 days, the TN removal rate (TNRR) and efficiency (TNRE) reached 15.1 g N/m^3^/h and 80%, respectively.

Thereafter (days 32–60), the TNRE decreased to 70% as the influent NH_4_^+^-N concentration increased, but the TNRR remained stable at approximately 0.26 kg N/m^3^/d, indicating that the studied CANON system had achieved steady-state nitrogen removal performance (Figure 1). Also, the ammonium oxidation rate (AOR) and the TN removal rate during the aeration stage (NRR) reached 21.2 g N/m^3^/h and 0.26 kg N/m^3^/day, respectively, and nitrite oxidation rate (NOR) was below 3 g N/m^3^/h, suggesting that the NOB proliferation had been controlled.

### 3.2. The CANON Performance Operated at Alternative Low and High Ammonium Concentrations

After a stable nitrogen removal performance was achieved in Phase high I, the CANON system adopted an alternative low and high ammonium concentration inflowing mode, that is, Phase low I and Phase high II (Figure 1).

#### 3.2.1. Operating at a Low Ammonium Concentration in Phase Low I

**Figure 2:**
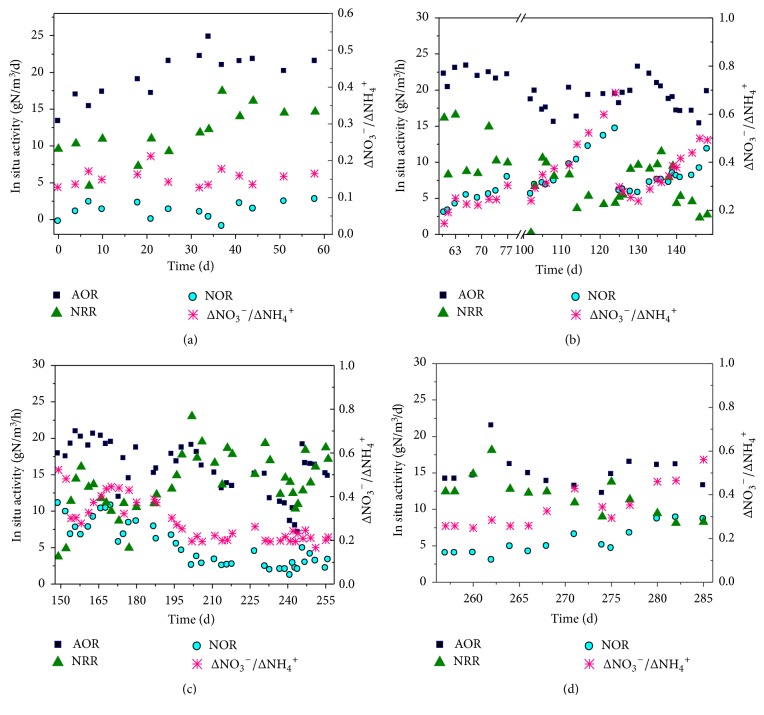
Changes in AOR, NOR, NRR, and ΔNO_3_^−^/ΔNH_4_^+^ ratio of the CANON system over the whole operational duration ((a) Phase high I; (b) Phase low I; (c) Phase high II; (d) Phase low II).

*(1) Variations in Nitrogen Removal Performance*. After the influent ammonium decreased, the nitrogen removal performance of the CANON system was stable firstly (days 61–74) and decreased thereafter (days 77–148) (Figure 1). Specifically, when the influent NH_4_^+^-N was decreased to 77 ± 4.5 mg/L on day 61, the TNRR immediately decreased from 0.26 kg N/m^3^/d (day 60) to 0.17 kg N/m^3^/d (day 61) with a decrease percentage of 32%. Luckily, the AOR was still stable at 22 g N/m^3^/h for about 10 days at low influent ammonium concentration, even being slightly higher than 21.2 g N/m^3^/h in Phase high I (Figures 2(a) and 2(b)). In contrast, NRR dropped to 11 g N/m^3^/h, being lower than 15.1 g N/m^3^/h of Phase high I, indicating that the low influent NH_4_^+^-N concentration had a greater impact on anammox reaction than on ammonia oxidation reaction.

Notably, NOR increased gradually to approximately 4.9 g N/m^3^/h during days 61–74 at a low ammonium influent (Figure 2(b)), suggesting that anammox bacteria could not compete with NOB for nitrite under the low influent NH_4_^+^-N concentration operation [6]. As the inhibitory threshold of FA for AOB and NOB is 8–120 mg N/L and 0.08–0.82, respectively [18], NOB are generally more sensitive to FA than AOB and can be outcompeted by AOB under a high ammonium environment (generally high FA as well). However, as shown in Table 1, at low NH_4_^+^-N concentrations, the competitive capacity of AOB for oxygen was not much greater than that of NOB due to the low FA (below 3 mg N/L) in Phase low I in this study. Nevertheless, during days 61–74 in Phase low I, the TNRE of the CANON was stable at 75% as a whole. Also, when compared with Phase high I, ΔNO_3_^−^/ΔNH_4_^+^ increased but was stable at approximately 0.23 (Figure 2(b)), indicating that the NOB abundance or activities were stable during these 14 days.

During days 74–124 (Phase low I), the nitrogen removal performance of the CANON continuously decreased (Figure 1). For instance, the TNRR and TNRE decreased to 0.05 kg N/m^3^/d and 23%, respectively. Although the AOR was still stable at 18 g N/m^3^/h, the NOR increased sharply to 14.6 g N/m^3^/h with the ΔNO_3_^−^/ΔNH_4_^+^ being high at 0.69 (Figure 2(b)). Also, the nitrate concentration in the effluent increased to 27.4 mg/L (Figure 1). This is possibly because anammox bacteria was not able to compete with NOB for nitrite at a low influent NH_4_^+^-N concentration, which untimely exposed anammox bacteria to a famine scenario.

It should be noted that the CANON reactor was shut down from days 78 to 101 because of the time controlling breakdown. Then the NOR increased sharply from 5.57 g N/m^3^/h on 102 d to 14.6 g N/m^3^/h on 124 d (Figure 2(b)). Considering that NOB and AOB are prone to colony in flocs, and anammox bacteria tends to be aggregated as granules, we meshed the activated sludge of the CANON system, to quickly recover the CANON performance. Specifically, 1 L mixed liquid was drawn from the CANON reactor, and the flocs were removed using a screen with 80 mesh. The left granules were poured into the CANON reactor again with 1 L shortcut nitrifying sludge (mainly containing AOB) from a shortcut nitration reactor in our laboratory.

After 7 days' recovery (day 130), the TNRR and TNRE increased to 0.17 kg N/m^3^/d and 64.5%, respectively (Figure 1). The AOR remained constant because of the low DO (0.2–0.4 mg/L) but NOR decreased by 60% from 14.6 g N/m^3^/h (day 124 in Phase low I) to 6 g N/m^3^/h (day 130). The NRR increased to 9.6 g N/m^3^/h and ΔNO_3_^−^/ΔNH_4_^+^ decreased to 0.24 with decreasing NOR (Figure 2(b)). This result suggests that it was suitable to washout NOB and recover the nitrogen removal performance in a short time through removing the flocs, as AOB and NOB are mainly colonized in flocs [15].

After day 130 in Phase low I, the CANON reactor deteriorated again (Figure 1). Notably, the NOR increased to 8 g N/m^3^/h on day 140 and to 11.8 g N/m^3^/h on day 148 (Figure 2(b)). Meanwhile, TNRE decreased to 36.8% and ΔNO_3_^−^/ΔNH_4_^+^ increased to 0.5 (Figure 2(b)), indicating that NOB had proliferated again and competed nitrite with anammox bacteria. It seems that controlling only DO at a low level could not sustain a steady shortcut nitrification [19].

**Figure 3:**
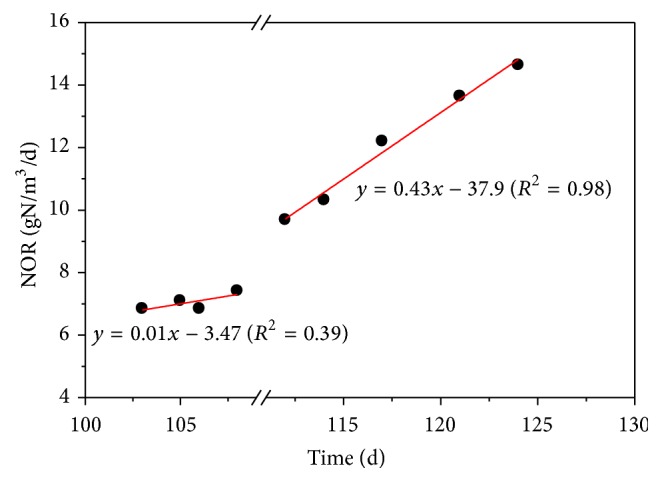
Linear fittings of NOR and time of the CANON reactor during the operation of days 103–108 and days 112–124 of Phase low I.

*(2) The NOR Variation Characteristics*. Remarkably, there was a linear relationship between NOR and operational days that NOR increased linearly with the operational days (Figure 3). When NOR was below 8 g N/m^3^/h, the NOR was gradually increased along with the operational days with a slope of 0.39. However, after NOR was beyond 8 g N/m^3^/h, the NOR sharply increased with a slope of 0.98 until the CANON SBR completely collapsed. It seems that the NOR should be controlled under 8 g N/m^3^/h to ensure the stable nitrogen removal performance of the studied CANON system. Thus, NOR was selected as an indicting parameter for CANON operated at a low ammonium concentration.

#### 3.2.2. Operating at a High Ammonium Concentration in Phase High II

On day 149, the influent ammonium concentration was increased to 240 ± 21 mg/L again to recover the CANON performance (Figure 1, Phase high II). The pH in the inflow was still controlled at 8.0, and the influent FA concentration was about 7–10 mg N/L. By improving the FA concentration, we expected to stress the NOB proliferation and recover the functional bacteria such as AOB and anammox bacteria in the studied CANON. Corresponding to the increased influent ammonium, the aeration time increased by 80% from 300 min to 540 min for one day with the unchanged DO concentration at 0.2–0.4 mg/L. Then, both the FA and DO concentrations set in Phase high II were favorable to inhibition of NOB [18, 20, 21].

*(1) The Stable Nitrogen Removal Performance at High Ammonium Concentration*. During day 149 to day 158 when operating at a high ammonium concentration of approximately 240 mg/L, the nitrogen removal performance of the CANON system continued to increase with the TNRR and NRR up to 0.25 kg N/m^3^/d and 16.1 g N/m^3^/h, respectively, on day 158 (Figures 1 and 2(c)). Correspondingly, the NOR and ΔNO_3_^−^/ΔNH_4_^+^ decreased to 6.7 g N/m^3^/h (below 8 g N/m^3^/h) and 0.28, respectively (Figure 2(c)), indicating that NOB had been effectively inhibited at the high ammonium concentration, and then anammox bacteria could compete with NOB for nitrite.

However, the nitrogen removal performance of the CANON system decreased from day 159 (Figure 1). Specifically, the NRR decreased to 10 g N/m^3^/h on day 170, and the NOR and ΔNO_3_^−^/ΔNH_4_^+^ increased to 10.7 g N/m^3^/h and 0.45, respectively (Figure 2(c)). These results illustrate that NOB might have been adapted to the high FA and proliferated again even at the high ammonia environment [22]. Our results are somewhat in contrast to those of Wang and Gao [23], who recovered the CANON system in 56 days from the excessive multiplication of NOB using simultaneous high ammonium and nitrite concentration in the inflow. The different results observed in this study with other studies are possible due to the fact that it was difficult to keep the high nitrite in the studied CANON system, as nitrite produced by AOB could be simultaneously or promptly be assumed by anammox bacteria in the CANON operational mode.

*(2) SRT Adjustment to Washout NOB*. Once the over proliferated NOB occurred in the CANON system, it is difficult to inhibit NOB due to the low decay rate of NOB [24]. This problem can be resolved by discharging the NOB sludge [8]. For example, in Strass WWTP (Austria), separation of AOB, NOB and anammox bacteria was handled by a hydrocyclone, and washing out NOB was effectively achieved by controlling of the selected SRT of AOB and anammox bacteria [25, 26]. Mimicking this case, from day 175, sludge discharging was adopted to the CANON system to control the SRT of approximately 60 d. Then, during days 175–202, the TNRR and TNRE increased to 0.29 kg N/m^3^/d and 63.5% (day 202) (Figure 1), respectively; the NOR decreased to 2.6 g N/m^3^/h with the effluent nitrate concentration decreased from 64.2 mg/L (day 175) to 45.6 mg/L (day 202) (Figures 1 and 2(c)). Also, the ΔNO_3_^−^/ΔNH_4_^+^ decreased to 0.2 on day 202, and NRR increased to 23 g N/m^3^/h (Figure 2(c)).

Although a part of NOB was washed out by the sludge discharging, the AOR also decreased due to the loss of the activated sludge. Consequently, the TNRR decreased gradually with further discharge of sludge. Specifically, the AOR and NRR decreased to 13.4 g N/m^3^/h and 17.8 g N/m^3^/h, respectively, on day 218 (Figure 2(c)). As shown in Figure S2, the MLVSS of the studied reactor decreased gradually due to over discharge of the sludge. As a result, the amount and activity of AOB and anammox bacteria decreased. However, because NOB was less abundant in the biomass than AOB and anammox bacteria, sludge discharging would lead to a lower percentage of NOB in the residual CANON system. Therefore, the NOR was still stable at 2.6 g N/m^3^/h (far below 8 g N/m^3^/h) and ΔNO_3_^−^/ΔNH_4_^+^ was also stable at 0.2 during days 202–218 (Figure 2(c)). To prevent the continued decrease in the TNRR, the sludge discharging was stopped on day 219 (Figure 1). With increasing MLVSS during days 219–256, the TNRR and TNRE were increased to 0.23 kg N/m^3^/d and 55% on day 256 (Figure 1), and the NOR and ΔNO_3_^−^/ΔNH_4_^+^ could be stable at 3.3 g N/m^3^/h and 0.2 (Figure 2(c)), respectively.

Taken together, our results show that high ammonium concentration (240 mg/L) and controlled SRT (60 day) could improve the performance of CANON system through decreasing NOB to a low abundance in 28 days (days 175–202).

#### 3.2.3. Stability of the CANON System in a Low Ammonium Concentration in Phase Low II

After day 257 (in Phase low II), the ammonium concentration was decreased to 61 ± 5.6 mg/L again (Figure 1) to examine the stability of CANON system at low ammonium concentrations. During day 257 to day 266 (approximately 10 days), the performance of the CANON system was stable. TNRR and TNRE were stable at 0.15 kg N/m^3^/d and 70% (Figure 1), respectively. However, after day 267, the CANON system deteriorated again; that is, the TNRR was lower than 0.1 kg N/m^3^/d and NOR increased to 8 g N/m^3^/h after day 279 (Figures 1 and 2(d)). The operational results during Phase low II confirmed that the system would deteriorate once NOR reached 8 g N/m^3^/h.

In summary, in Phase low I (day 61–74) and Phase low II (day 257–266) the CANON system could be stably operated for approximately 10 days at low ammonium concentrations (60 mg/L) with a relatively high TNRE (70%) and low effluent N concentrations (5 mg NH_4_^+^-N/L and 20 mg TN/L) (Figure 1).

#### 3.2.4. Microbial Composition and Structure Variations with Cyclic Low and High Influent Ammonium

**Figure 4:**
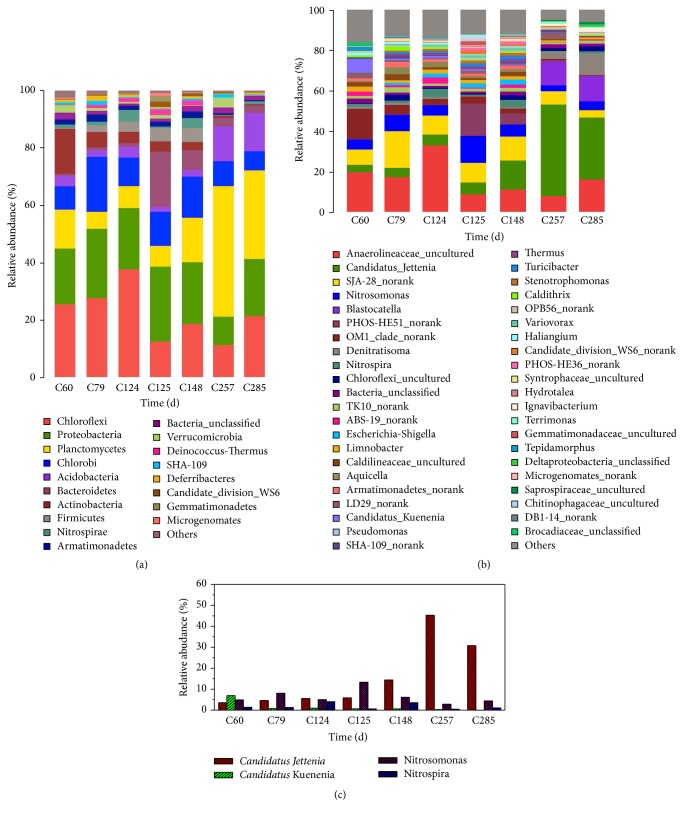
The microbial community taxonomic compositions in the studied CANON system: (a) at the phylum level; (b) at the genus level; (c) changes in the relative abundance of* Candidatus* Jettenia,* Candidatus* Kuenenia,* Nitrosomonas,* and* Nitrospira*.

*(1) Bacterial Community Composition*. The composition of the bacterial communities in the CANON system was analyzed by 16S rDNA high-throughput sequencing. At phylum level, the CANON system was dominated by Chloroflexi, Proteobacteria, Planctomycetes, and Chlorobi (Figure 4(a)). Anammox bacteria and AOB, the functional bacteria in the CANON system, were affiliated to Planctomycetes and Proteobacteria, respectively. Chloroflexi and Chlorobi were also extensively detected in other anammox systems [27], and Chloroflexi could provide structure support for sludge granulation using the decayed anammox biomass [28, 29].

The N-related bacteria were* Nitrosomonas*-affiliated AOB,* Nitrospira*-affiliated NOB, and “*Candidatus *Jettenia” anammox bacteria (Figure 4(b)). Chu et al. [30] also found that “*Candidatus* Jettenia” and* Nitrosomonas* were the dominant functional bacteria in their CANON system treating high ammonium wastewater (500 mg N L^−1^), with the relative abundances 16.8% and 20.1%, respectively. It should be noted that there also existed* Denitratisoma*-affiliated denitrifying bacteria in the CANON system.* Denitratisoma* was reported to be able to use 17b-oestradiol as the sole carbon source and energy and electron donor to reduce nitrite to nitrous oxide [31].

*(2) The Variations of N-Transformation Microorganisms*. In order to elucidate the variations in the abundances of the N-related bacterium,* Candidatus* Jettenia,* Candidatus* Kuenenia,* Nitrosomonas, *and* Nitrospira *were plotted in Figure 4(c). When the CANON system was stable in Phase high I (day 60),* Candidatus *Jettenia and* Candidatus *Kuenenia were the dominant anammox bacteria with the relative abundances of 3.56% and 6.9%, respectively. However, when the ammonium concentration decreased in Phase low I,* Candidatus *Jettenia outcompeted* Candidatus *Kuenenia and became the main anammox genera (day 79). Specifically, from day 124 to day 148 in Phase low I, the relative abundance of* Candidatus *Jettenia increased from 5.91% to 14.4%. This is primarily because the increased AOB amount and decreased NOB amount after sludge changing caused the increased nitrite concentration.

After being cultivated during Phase high II,* Candidatus *Jettenia was still the dominant anammox bacteria (days 257–285), while the relative abundance of* Candidatus *Kuenenia was at an extremely low level (0.25% and 0.02% on days 257 and 285, respectively). For example, the relative abundance of* Candidatus *Jettenia increased from 14.4% (day 148, Phase low I) to 45.32% (day 257, Phase high II). Obviously, a high ammonium concentration had favored to enrich anammox bacteria. As each genus of anammox bacteria has its own special ecological niche [32], the higher relative abundance of* Candidatus *Jettenia than that of* Candidatus *Kuenenia indicates that the present experimental condition was more suitable for* Candidatus *Jettenia.

As shown in Figure 4(c), on days 124 (Phase low I), 148 (Phase low I), and 285 (Phase low II), the relative abundance of* Nitrospira* (NOB) increased slightly because of the low ammonium concentration. However, after the sludge changing on day 124, the relative abundance of* Nitrospira *decreased from 4.01% (day 124) to 0.57% (day 125), and* Nitrosomonas *increased from 5.03% to 13.32%. The relative abundance of* Nitrospira *also decreased from 3.55% (day 148) to 0.43% (day 257) after being cultivated at a high ammonium concentration in Phase high II, suggesting that it was suitable to enrich AOB and inhibit NOB using sludge changing and a high ammonium concentration.

Compared with Phases high I and low I, Phases high II and low II seem to be more robust in the nitrogen removal performance (Figure 1), possibly because the relative abundance of anammox bacteria was high in Phases high II and low II (Figures 4(b) and 4(c)). Our results suggest that replenishing anammox bacteria biomass into the CANON system could be an alternative strategy for stabilization of anammox treatment performance.

### 3.3. The Recommended Operation Strategy for Practical Operation of CANON Systems

According to our experimental results, the alternative low and high ammonium influent regime was feasible for CANON system to treat a part of low ammonium wastewater. It is recommended alternatively to operate CANON system at low ammonium concentration for 10 days and at high ammonium concentration for 28 days. Also, the NOR and sludge age (SRT), as two important parameters, were recommended to be below 8 g N/m^3^/h and approximately 60 d in the present CANON systems.

The proposed strategy can be realized if WWTPs have sludge digestion unit, from which the higher ammonium influent can be supplied. Also, several parallel CANON SBR units are required so that when a series CANON SBRs treat mainstream wastewater, other series can treat sidestream wastewater (i.e., sludge digestion supernatant) for enhancement of AOB and anammox bacteria, and inhibition of NOB. By this way, CANON system can treat nitrogen containing wastewater continuously. But to use this operational regime successfully in mainstream CANON system, the difference between actual ammonium concentration in real WWTPs and our experiment must be considered. Further research should be focused on improving the proportion of the low ammonium concentration treatment duration, and overcoming low temperature in real municipal wastewater. Our strategy hopes to help opening a new possibility for CANON processes used in municipal wastewater (mainstream wastewater) treatment.

## 4. Conclusions

An alternative low and high ammonium influent regime was proposed and investigated to keep CANON stable when treating low ammonium wastewater. Alternatively operating at a low ammonium concentration for 10 days and at a high ammonium concentration for 28 days was feasible for CANON to treat low ammonium wastewater. NOR and sludge age, as two important parameters, should be controlled to maintain a stable operation. NOR should be kept under 8 g N/m^3^/h to prevent CANON deterioration. To use CANON in mainstream successfully, further studies are needed to shorten the duration of operating at high ammonium concentrations and overcome low temperature.

## Figures and Tables

**Table 1:** Operating parameters of the CANON system.

Phase	High I	Low I	High II	Low II
Periods (d)	0–60	61–148	149–256	257–285

Cycle time (h)	8	4	8	4

Influent FA (mg N/L)	5–7	<3	7–10	<3

Influent NH_4_^+^-N (mg/L)	196 ± 18	77 ± 4.5	240 ± 21	61 ± 5.6

Volumetric exchange ratio	0.5			

DO (mg O_2_/L)	0.2–0.4	0.2–0.4	0.2–0.4	0.4–0.6

Temperature (°C)	30	30	30	30

Aeration mode	3 aeration and anoxic stages in one SBR cycle (35 min/105 min, respectively)	50-min aeration stage in one SBR cycle	3 aeration and anoxic stages in one SBR cycle (60 min/60 min, respectively)	50-min aeration stage in one SBR cycle

## Supplementary Materials

Supplementary Materials Figure S1: the anammox granule of seeding CANON sludge (A); seeding CANON sludge (B); anammox granule after sieving on day 124 (C); flocs after sieving on day 124 (D). Figure S2: changes in the MLSS, MLVSS and ΔNO_3_^−^/ΔNH_4_^+^ ratio over the operation of Phase high II. Figure S3: rarefaction curve. Table S1: microbial community richness and diversity index of sludge samples.
