# Supplementary material for: Keeping a Completely Autotrophic Nitrogen Removal over Nitrite System Effective in Treating Low Ammonium Wastewater by Adopting an Alternative Low and High Ammonium Influent Regime
Source: Biomed Res Int. 2018 Apr 17;2018:9536761. doi: 10.1155/2018/9536761 (PMC5932508; doi:10.1155/2018/9536761)
Supplement: Supplementary Materials — Figure S1: the anammox granule of seeding CANON sludge (A); seeding CANON sludge (B); anammox granule after sieving on day 124 (C); flocs after sieving on day 124 (D). Figure S2: changes in the MLSS, MLVSS and ΔNO3−/ΔNH4+ ratio over the operation of Phase high II. Figure S3: rarefaction curve. Table S1: microbial community richness and diversity index of sludge samples. [file 9536761.f1.pdf]

## Supporting Information

# Keeping a completely autotrophic nitrogen removal over nitrite (CANON) system effective in treating low ammonium wastewater adopting an alternative low and high-ammonium influent regime

Chang Qinglong, Weigang Wang, Jie Chen, Yayi Wang*

State Key Laboratory of Pollution Control and Resources Reuse, College of Environmental Science and Engineering, Tongji University, Siping Road, Shanghai 200092, P. R. China

*Corresponding author. E-mail:

wyywater@126.com; yayi.wang@tongji.edu.cn

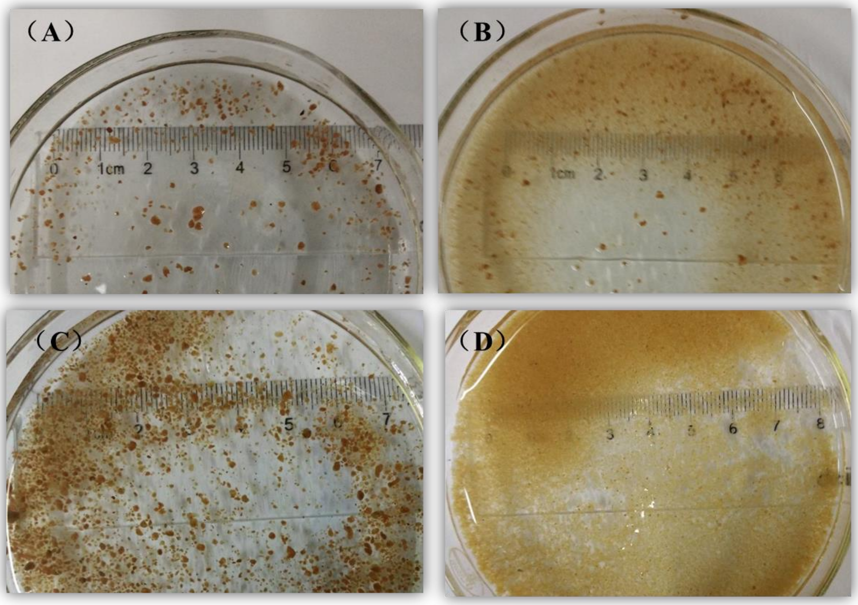


**Fig. S1.** The anammox granule of seeding CANON sludge (A); seeding CANON sludge (B); anammox granule after sieving on day 124 (C); flocs after sieving on day 124 (D).

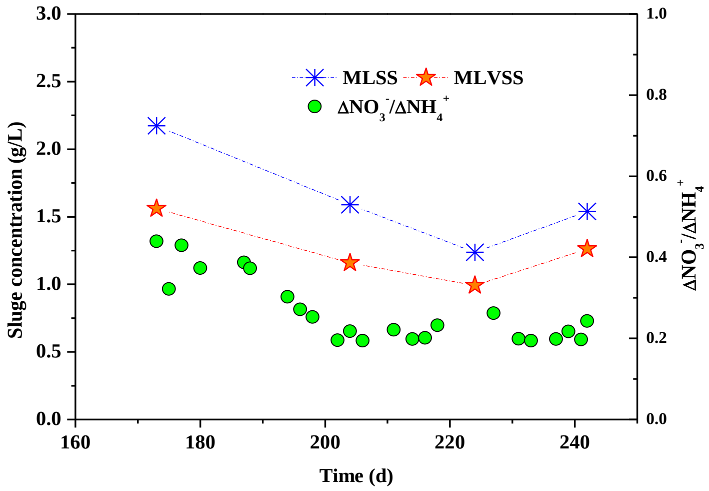


**Fig. S2** Changes in the MLSS, MLVSS and $\Delta NO_3^-/\Delta NH_4^+$ ratio over the operation of Phase high II.

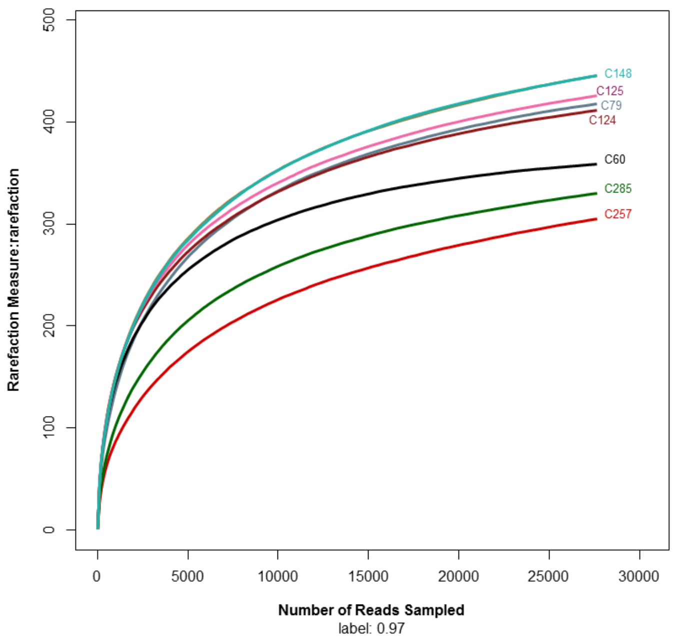


**Fig. S3** Rarefaction curve.

**Table S1** Microbial community richness and diversity index of sludge samples

| Sludge sample No. | Sequence Reads | OTU numbers | ACE Index | Chao Index | Shannon Index | Simpson Index | Coverage |
|---|---|---|---|---|---|---|---|
| C60 | 27631 | 359 | 378 | 378 | 4.03 | 0.0451 | 0.9986 |
| C79 | 27631 | 426 | 482 | 482 | 4.07 | 0.0489 | 0.9972 |
| C124 | 27631 | 411 | 453 | 447 | 4.06 | 0.0441 | 0.9977 |
| C125 | 27631 | 445 | 507 | 530 | 3.92 | 0.0563 | 0.9969 |
| C148 | 27631 | 445 | 506 | 510 | 4.02 | 0.0474 | 0.9970 |
| C257 | 27631 | 305 | 377 | 382 | 2.64 | 0.2268 | 0.9971 |
| C285 | 27631 | 330 | 374 | 383 | 3.09 | 0.1277 | 0.9976 |
